# Genome-wide association study reveals novel candidate genes for litter size in Markhoz goats

**DOI:** 10.3389/fvets.2022.1045589

**Published:** 2022-11-23

**Authors:** Peyman Mahmoudi, Amir Rashidi, Anahit Nazari-Ghadikolaei, Jalal Rostamzadeh, Mohammad Razmkabir, Heather Jay Huson

**Affiliations:** ^1^Department of Animal Science, Faculty of Agriculture, University of Kurdistan, Sanandaj, Iran; ^2^Department of Animal Breeding and Genetics, Swedish University of Agricultural Sciences, Uppsala, Sweden; ^3^Department of Animal Science, Cornell University, Ithaca, NY, United States

**Keywords:** genome-wide association study, litter size, Markhoz goat, reproduction, prolificacy

## Abstract

**Introduction:**

The Markhoz goat is the only breed that can produce high-quality fiber called mohair in Iran; however, the size of its population has faced a dramatic decline during the last decades, mainly due to the reluctance of farmers to rear Markhoz goats caused by a reduction in goat production income. Litter size at birth (LSB) and weaning (LSW) are two economically important reproductive traits for local goat breeders and have the potential of increasing the population growth rate. The present study was aimed to identify possible genomic regions that are associated with LSB and LSW in Markhoz goats using a genome-wide association study (GWAS).

**Methods:**

To this end, 136 Markhoz goats with record(s) of kidding were selected for GWAS using the Illumina Caprine 50K bead chip. The individual breeding values (BV) of available LSB and LSW records estimated under an animal mixed model were used as the dependent variable in the GWAS, thereby incorporating repeated categorical variables of litter size.

**Results:**

Four SNPs on chromosomes 2, 20 and 21 were identified to be significantly associated (FDR *p* < 0.05) with LSB after multiple testing correction under a Bayesian-information and Linkage-disequilibrium Iteratively Nested Keyway (BLINK) model. Least-square analysis was performed to investigate the effects of detected genotypes on LSB. Ultimately, the GWAS results introduced six candidate genes, including *GABRA5, AKAP13, SV2B, PPP1R1C, SSFA2* and *TRNAS-GCU* in a 100 kb adjacent region of the identified SNPs. Previous studies proposed functional roles of *GABRA5* and *AKAP13* genes in reproductive processes; however, the role of other candidate genes in reproduction is not clear.

**Conclusion:**

These findings warrant further investigation for use in marker-assisted selection programs in Markhoz goats.

## Introduction

Throughout history, goats have been a primary production species for mankind due to their ability to withstand variable and harsh environmental conditions and their desirable production of meat, milk, fiber, and skin. Improving economic traits in goats, such as prolificacy and viability, can be very profitable for rural people in countries with low-quality grazing lands, where goat farming is one of the primary sources of income.

Markhoz goats are the only mohair producing breed in Iran with different coat colors such as white, black, and various shades of brown (1). The population size of the Markhoz goat underwent a considerable decrease during the last two decades (2), such that only about 1,000 head remain in their main native habitat in western and northwestern Iran. The main reasons for the decreased population of the indigenous Markhoz goat include changes in the management system for how animals are reared and the generally low income gained by goat farming in local regions. Hence, increasing the number of kids born per kidding and subsequently increasing the total income through the selling of kids, fiber, and meat may encourage ranchers toward goat production.

Litter size at birth (LSB) and litter size at weaning (LSW) are two reproductive traits that are known to be controlled by several underlying genes in goats (3). Specifically, genetic variants with significant effects on LSB have been identified in genes including, *GDF9, BMP15, GnRH1, KISS1, KITLG, NGF, POU1F1, PRLR* and promoter of *miR-9* gene in various breeds of goats (4–11). However, there may be other genes that affect LSB and LSW that have remained unknown. Nowadays, with the development and availability of SNP genotyping technologies, conducting genome-wide association studies (GWASs) and detecting candidate genes and genetic variants that may have a significant association with economic traits, have become much easier and faster. In this regard, many GWASs have been conducted using the Illumina Caprine 50K beadchip [Illumina Inc., San Diego, CA (12)] for various economically important traits in different goat breeds including coat color and mohair traits (13), body morphological traits (14), conformation and milk yield (15), and resistance to nematodes (16). Similarly, multiple researchers have conducted GWAS on liter size at birth in sheep (17–19). However, there is only one GWAS for the number of kids alive per kidding in goat (20) and no GWAS for the number of kids alive till weaning in sheep or goat.

Detection of significantly associated SNPs with LSB and LSW in goat would lead to enhanced efficacy of animal selection in breeding strategies by reducing the cost and time required to raise and phenotypically characterize animals as they mature. As complex traits, LSB and LSW are known to be controlled by many genes with variants having a small effect. Hence, the possibility of incorporating genetic variants into selection strategies will likely accelerate the rate of improvement of such reproductive traits as compared to traditional phenotypic selection. The purpose of this study was to conduct GWAS to detect possible genomic regions and variants associated with LSB and LSW in Markhoz goats, with the potential of applying results in genomic selection.

## Materials and methods

### Animals and phenotypes

All female goats (*n* = 184) existing at the Markhoz Goat Performance Testing Station in Sanandaj, Kurdistan, Iran, were selected for inclusion in the study. The herd is reared under a semi-intensive management system, in which animals graze on natural pastures from spring to early autumn and fed a diet consisting of alfalfa and wheat straw for the rest of the year. At the age of 16–18 months, does are mated for the first time. The kidding season starts in late winter and ends in early spring. Litter size at birth (LSB) and litter size at weaning (LSW) were the two reproductive traits evaluated. LSB described the number of live kids born to the doe. LSW described the number of live kids at weaning for each doe, typically evaluating kids at 22–27 weeks old. LSB and LSW were categorical variables potentially repeated per doe as they aged and had subsequent litters.

### Statistical analyses

#### Prediction of breeding values

Predicted breeding values (PBV) for LSB and LSW were generated for use in the GWAS to capture the repeated categorical values of LSB and LSW. A total of 3,410 litter size records for the Markhoz goats were collected from 1994 to 2019 for use in predicting breeding values. Accuracy of EBVs is based on the amount of performance information available on the animal and its close relatives. Selection using EBVs is more accurate, especially for low heritable traits like litter size, which allows for faster genetic gain compared to mass selection using phenotypes. Breeding values have the advantage that they are free of systematic environmental effects on measured phenotypes, as these effects are considered in the statistical model used for the estimation of EBVs. Additionally, they reflect the genetic makeup more accurately because they do not solely rely on own records but include information from all measured relatives. The pedigree file included 5,396 animals with 1,533 dams and 252 sires. The number of founders, individuals with progeny, and individuals without progeny were 343, 1,785 and 3,611, respectively. Breeding values for each individual was estimated, applying a repeatability threshold animal model using ASReml 2.0 (21) as follows:

$$\begin{array}{l} y=Xb+Za+Wpe+e \end{array}$$

where y is a vector of phenotypic value for LSB/LSW, b, a, pe and e are vectors of fixed effects including year of kidding (2010–2019), age of dam (2–9 years) and parity (1–7), random animal effects, random permanent environmental effects and random residual effects, respectively. X, Z, and W are design matrices that relate records to fixed, animal and permanent environmental effects, respectively.

#### Genotyping and quality control

All animal procedures were approved by the Cornell University Institutional Animal Care and Use Committee prior to sampling (protocol #2014-0121). Vacutainer tubes containing K_2_EDTA as an anticoagulant were used to collect whole blood (5 ml) samples from the jugular vein of goats. Samples were immediately stored at −20°C until DNA extraction. A standard Phenol-Chloroform DNA extraction method was utilized for extracting genomic DNA (22). The Illumina Caprine 50K beadchip (Illumina, Inc., San Diego, CA, United States), including 53,353 SNPs, was used for genotyping samples (VHL Genetics, Wageningen, Netherlands). Golden Helix SVS v8.3.4 (Golden Helix, Bozeman, MT, United States) software was used for quality control process as follows: (1) 624 SNPs were removed for a call rate less than 0.9; (2) 2,540 SNPs with a minor allele frequency less than 0.03 were excluded; (3) 810 SNPs were not assigned to a genomic location; thus they were removed; (4) five samples were removed for a genotyping call rate less than 0.9; and (5) three samples with an estimated identity-by-state (IBS) score greater than 0.9 were removed to eliminate the possible effects of substantial relatedness between individuals on the overall results. Furthermore, 39 samples were excluded because they had no history of kidding in the data set. After the quality control process, 49,764 SNPs and 136 animals remained for GWAS.

#### Genome-wide association studies

The GAPIT v3.0 R package was used for investigating the association between genomic regions and PBVs for LSB and LSW as phenotypes. Several models including General Linear Model (GLM), Mixed Linear Model (MLM), Multi Locus Mixed Model (MLMM), Compressed Mixed Linear Model (CMLM), Enriched Compressed Mixed Linear Model (ECMLM), Factored spectrally transformed Linear Mixed Model (Fast-LMM), Settlement of MLM Under Progressively Exclusive Relationship (SUPER), Fixed and random model Circulating Probability Unification (FarmCPU), Efficient Mixed-Model Association (EMMA), Efficient Mixed Model Association eXpedited (EMMAX) and BLINK were performed to find the best model for fitting PBV LSB and LSW data (**Figure 2**). The BLINK (23) and FarmCPU (24) models showed less deviation from expectation in the Q-Q plots than other models. Despite FarmCPU identifying more significantly associated SNPs in the model comparison, BLINK was selected for the final GWAS because it controlled both false positives and false negatives effectively, showing a sharp upward deviated tail and a straight line close to the 1:1 line. In the BLINK method, markers in LD (*r*^2^ > 0.7) with the most significant marker are excluded from the analysis and the maximum likelihood of a random effect model is approximated by using the Bayesian Information Content (BIC) of a fixed-effects model. By applying that, the most significant markers will be selected among all markers that remained after LD exclusion and then used as cofactors in the model to test all markers across the genome. Because of the abovementioned reasons, the BLINK method had improved statistical power and better control on false positives in comparison with kinship-based methods (23). In the present study, the first 10 PCs explained about 23% of stratification, of which 12% was explained by the first three PCs. For considering population structure and avoiding biases due to population stratification in the present GWAS, only the first three PCs were included as covariates in the model, because no difference observed in the results when 4–10 top PCs (explained about 11% of stratification) were included in the analyses.

Significance of marker association was determined using a false discovery rate (FDR) adjusted *p*-value of less than 0.05. The ggplot R package was used to generate Q–Q and Manhattan plots (25). The genomic heritability was estimated using LDAK software v5.1 (26).

#### Least-square analysis and correlations between studied traits

Least-square analyses were conducted to investigate the negative/positive effects of detected genotypes on studied traits using PROC MIXED of SAS v8.2 software (27). Phenotypic and genotypic correlations between LSB and LSW were estimated using CORR procedure of SAS v8.2 and GCTA software tool (28), respectively.

#### Identifying candidate genes

A region with a distance of 100 kbp up-stream and 100 kbp down-stream of significant SNPs was explored to detect the nearest gene using *Capra hircus* ARS1 assembly (29) in the NCBI database. The LD decay pattern of the Markhoz goat population was estimated and the explored distance was selected based on the intersection point between the LD line and the *r*^2^ threshold determined the LD decay value (*r*^2^ < 0.1). Finally, gene databases, such as Genecards, National Center for Biotechnology Information (NCBI), Gene Ontology (GO) and Kyoto Encyclopedia of Genes and Genomes (KEGG) were scrutinized to find the functions and pathway of identified genes.

## Results

### Descriptive statistics and predicted breeding values

**Figure 1:**
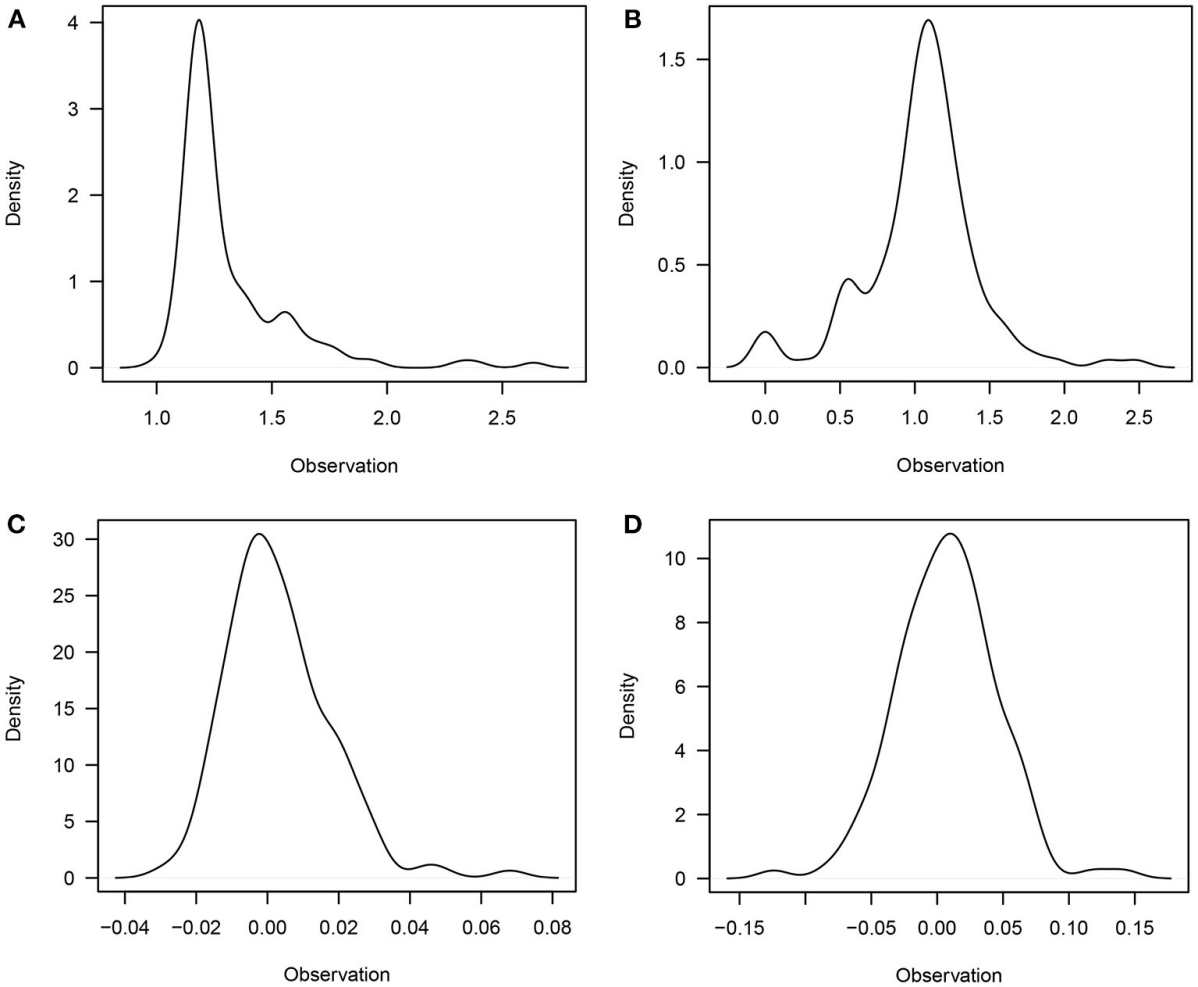
The distribution of litter size at birth **(A)**; litter size at weaning **(B)**; PBVs for litter size at birth **(C)**; and PBVs for litter size at weaning **(D)**.

The descriptive statistics and predicted breeding values for LSB and LSW traits are presented in Table 1. The studied population had an average LSB of 1.16, while only 0.99 of them remained alive at the age of weaning (LSW). The mean PBV for LSB and LSW was 0.0031 and 0.0073, respectively. Furthermore, the distribution of studied traits is depicted in Figure 1.

**Table 1:** Descriptive statistics and estimated breeding values for litter size at birth and litter size at weaning in the Markhoz goat.

**Statistic**	**LSB**	**LSB PBV**	**LSW**	**LSW PBV**
*n*	137	5,396	137	5,396
Mean (± SD)	1.16 (± 0.37)	0.0031 (± 0.0146)	0.99 (± 0.50)	0.0073 (± 0.0381)
Min	1	−0.029	0	−0.124
Max	3	0.068	2	0.142

LSB, litter size at birth; PBV, predicted breeding value; LSW, litter size at weaning.

### Genome-wide association studies

**Figure 2:**
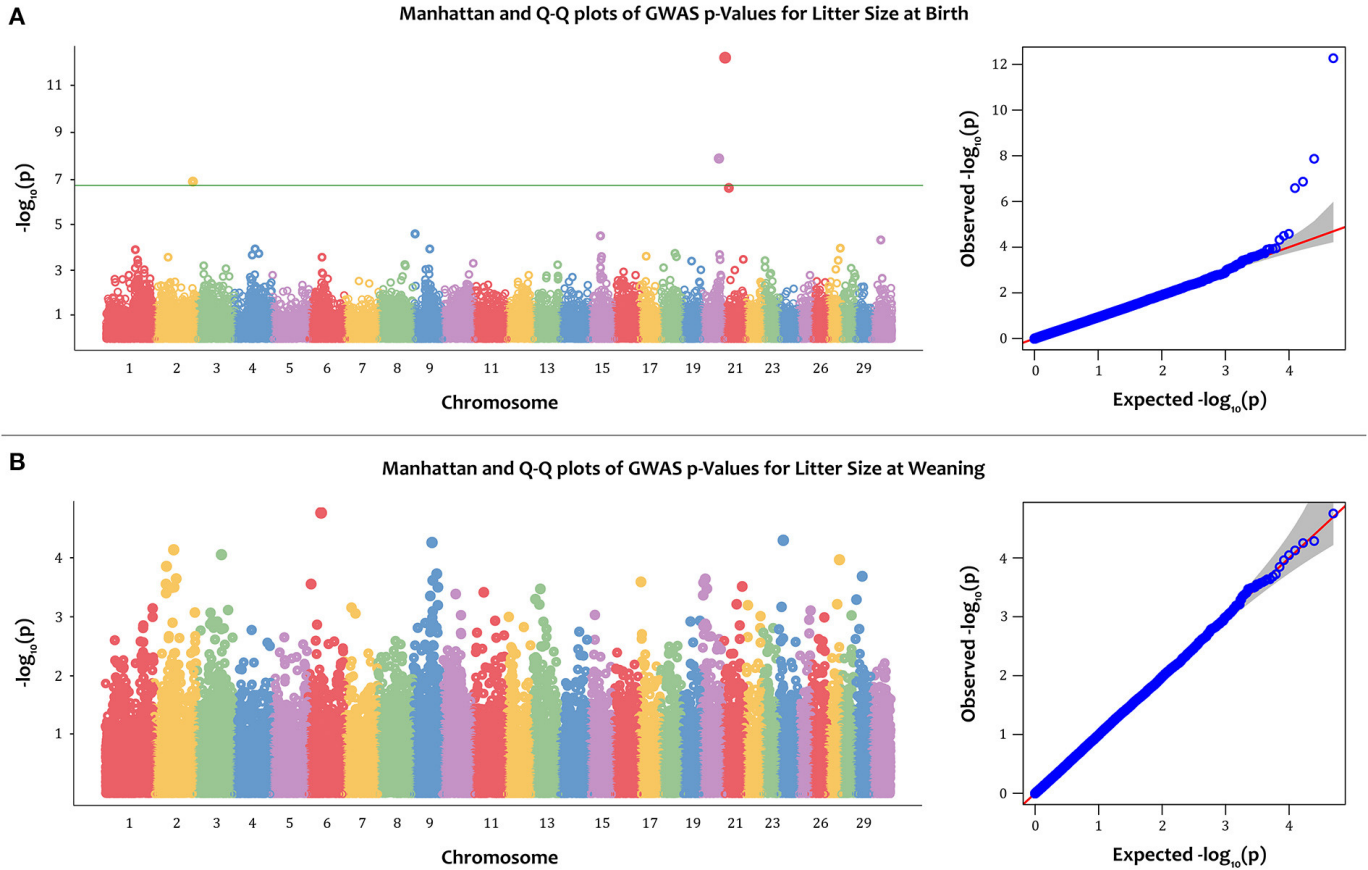
The Manhattan and quantile–quantile (Q–Q) plots of -log 10 (*p*-value) for litter size at birth (LSB) **(A)**; and litter size at weaning (LSW) **(B)** in the Markhoz goats using the BLINK GWAS model. The green horizontal line indicates the FDR adjusted *p*-value threshold of 0.05.

**Figure 3:**
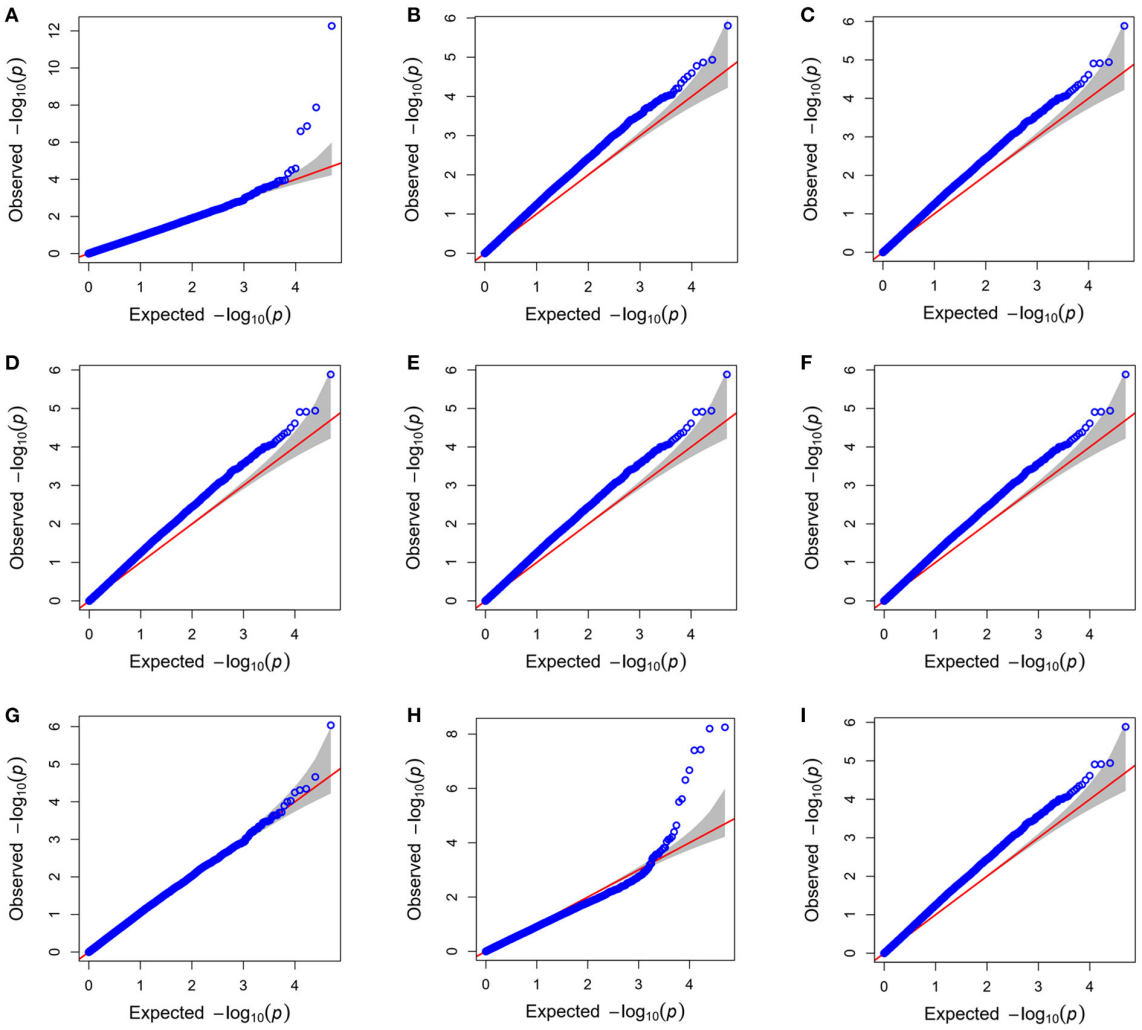
The Q–Q plot for litter size at birth (LSB) fitting Bayesian-information and Linkage-disequilibrium iteratively nested keyway (BLINK) **(A)**; general linear model (GLM) **(B)**; mixed linear model (MLM) and multi locus mixed model (MLMM) **(C)**; compressed mixed linear model (CMLM) **(D)**; enriched compressed mixed linear model (ECMLM) **(E)**, factored spectrally transformed linear mixed model (Fast-LMM) **(F)**; settlement of MLM under progressively exclusive relationship (SUPER) **(G)**; fixed and random model circulating probability unification (FarmCPU) **(H)**; and efficient mixed-model association (EMMA) and efficient mixed model association eXpedited (EMMAX) **(I)** GWAS models.

The Manhattan and Q-Q plots for PBV of LSB and LSW are depicted in Figure 2. Furthermore, the Q-Q plot for litter size at birth fitting other models is indicated in Figure 3. Four significantly associated SNPs on chromosome 2 (rs268267345, unadjusted *p*-value = 1.35e−07), chromosome 20 (rs268258357, unadjusted *p*-value = 1.33 e−08) and chromosome 21 (rs268288690, unadjusted *p*-value = 5.41e−13; rs268256209, unadjusted *p*-value = 2.56e−07) were identified for PBV LSB (Figure 2A). The FDR adjusted *p*-values for the identified SNPs were 0.002, 0.0003, 2.69e−08 and 0.003, respectively. The Q–Q plot for LSB showed a very slight genomic inflation factor with λ_*GC*_ = 1.01. In contrast, no significantly associated SNPs were found for PBV LSW after multiple testing correction under the various models tested for this trait. The detailed information, including location, alleles, and p-values, for significantly associated SNPs on the PBV LSB trait is provided in Table 2. Considering that the distance between the significantly associated SNPs from their neighboring SNPs was farther than 500 kb and the BLINK model uses a minimum distance of 300 kb for exclusion of markers, linkage disequilibrium analysis was not performed.

**Table 2:** Genome-wide association study identifies four SNPs significantly associated with the predicted breeding value of litter size at birth (LSB) in Markhoz goats.

**Chr**	**SNP name**	**SNP id**	**Pos (bp)**	**Alleles**	**Unadj-P**	**FDR**
2	snp35221-scaffold422-519219	rs268267345	121791230	A/G	1.35e−07	0.002
20	snp25995-scaffold269-1405685	rs268258357	55321621	A/G	1.33e−08	0.0003
21	snp57162-scaffold91-2213872	rs268288690	3499131	A/G	5.41e−13	2.69e−08
21	snp23799-scaffold240-558706	rs268256209	15409777	A/G	2.56e−07	0.003

Chr, chromosome number; Pos, chromosome position; Unadj-P, unadjusted *p*-value; FDR, false discovery rate.

### Least-square analysis of identified genotypes for LSB and phenotypic and genotypic correlations

The results of the least-square analyses for LSB are provided in Table 3. Results show that markers rs268267345, rs268258357, and rs268288690 in the genome of the Markhoz goats significantly resulted in increased litter size in goats having one or two mutated alleles. In contrast, rs268256209 SNP has a significant negative effect on LSB in goats that carry two G alleles.

**Table 3:** Genotypic frequency and least-square mean ± standard error of litter size at birth for identified SNPs in the Markhoz goats.

**SNP id**	**Genotype**	***N***	**LSMean^1^ ±SE**	***p*-value**
rs268267345	AA	65	1.19^b^ ± 0.04	0.048
	AG	58	1.25^ab^ ± 0.04	
	GG	14	1.35^a^ ± 0.07	
rs268258357	AA	115	1.20^c^ ± 0.03	<0.0001
	AG	21	1.31^b^ ± 0.05	
	GG	1	1.82^a^ ± 0.16	
rs268288690	AA	115	1.17^b^ ± 0.04	<0.0001
	AG	20	1.37^a^ ± 0.05	
	GG	2	1.48^a^ ± 0.15	
rs268256209	AA	33	1.30^a^ ± 0.05	0.001
	AG	74	1.24^a^ ± 0.04	
	GG	30	1.10^b^ ± 0.05	

1 Different letters indicate a statistically significant difference between genotypic groups.

Three genotypes of each locus are compared to each other.

The phenotypic correlation between LSB and LSW was 0.697 (*P* < 0.01). Furthermore, analysis of the genotypic correlation between these traits showed a strong genetic relationship of 0.725 (*P* < 0.01).

### Estimated genomic heritability

The estimated heritability using recorded data and pedigree for LSB and LSW of the entire population was 0.018 and 0.32e−6, respectively. However, the genomic heritability for studied traits was 0.011 and 0.21e−7, respectively. The detailed information for variance components of LSB and LSW traits is presented in Table 4.

**Table 4:** Estimation of variance components and genetic parameters for litter size at birth (LSB) and litter size at weaning (LSW) in the Markhoz goats.

**Trait**	** $\sigma_{a}^{2}$ **	** $\sigma_{pe}^{2}$ **	** $\sigma_{e}^{2}$ **	***h*^2^(±SE)**	** $h_{G}^{2}$ **	**R**
LSB	0.0024	0.49e−7	0.1278	0.019 (±0.028)	0.011	0.018
LSW	0.82e−7	0.0140	0.2421	0.32e−6 (±0.14e−6)	0.21e−7	0.055

$\sigma_{a}^{2}$, additive genetic variance; $\sigma_{pe}^{2},$ permanent environmental variance; $\sigma_{e}^{2},$ residual variance; *h*^2^, whole population heritability; $h_{G}^{2}$, genomic heritability; R, repeatability.

### Detected candidate genes

Six candidate genes were found within the 100 kb windows up- and down-stream of the identified SNPs located on the genome of *Capra hircus* in the NCBI database (ARS1 assembly, accession number: GCF_001704415.1). The detected genes harboring SNPs, their distance from SNPs and the roles of each gene are presented in Table 5.

**Table 5:** Genes within 100 kb distance from identified significant SNPs and their description.

**SNP ID**	**Candidate gene**	Distance^*^	**Gene description**
rs268267345	*PPP1R1C*	Intron 2	Protein phosphatase 1 regulatory inhibitor subunit 1C
	*SSFA2*	−98,584	Sperm-specific Antigen 2
rs268258357	*TRNAS-GCU*	−71,861	Transfer RNA Serine (Anticodon GCU)
rs268288690	*GABRA5*	Intron 6	Gamma-aminobutyric acid type a receptor subunit Alpha5
rs268256209	*SV2B*	−41,296	Synaptic vesicle glycoprotein 2B
	*AKAP13*	+99,644	A-Kinase anchoring protein 13

* The distance from identified SNP.

## Discussion

In Iran, the Markhoz goat is the only breed that can produce mohair in black, gray, white and varying shades of brown color. The fiber obtained from the Markhoz goat is both culturally and socially important for Kurdish people costumes (30). Thus, the decreasing population size of this valuable breed could be mitigated by using breeding programs incorporating genetic markers affecting reproductive traits such as litter size at birth (LSB). This is the first study on litter size traits in the Iranian goats at a genome-wide scale which can be employed as a practical tool to identify novel genetic markers that may influence litter size in the Markhoz and other goat breeds.

For both traits, the first three principal components (PCs) were included in the model to correct for population structure. A total of four SNPs, found on chromosomes 2, 20, and 21, were significantly associated with the PBV of LSB (Figure 2A). The Q–Q plots showed that observed versus expected data was well aligned, indicating minimal population stratification affecting the model (λ_*GC*_ = 1.01). In contrast, the results of the GWAS failed to reveal significant association with the PBV of LSW (Figure 2B).

The most significantly associated variant (rs268288690) with the PBV of LSB is located within the *GABRA5* gene. *GABRA5* encodes a 462 amino acid protein of the *GABA* receptors family known as *GABA-A*. It has been suggested that *GABA* acts at *GABA-A* receptors in the central and peripheral nervous systems as the major inhibitory neurotransmitter (31). Watanabe et al. (32) investigated the role of *GABA* in the regulation of GnRH neurons. They reported that in the course of fetal development, *GABA* is involved in the regulation of GnRH neuron migration from the olfactory placodes into the forebrain. They also stated that negative and positive feedback of estradiol are mediated by *GABA*, and there is a significant correlation between these feedbacks and frequency of *GABA* transmission to GnRH neurons. In a recent study, Di Giorgio et al. (33) demonstrated that *GABA-A* and *GABABRs* interact with kisspeptin (a protein encoded by the *Kiss1* gene) in the regulation of reproductive processes. For instance, *GABA* increases *Kiss1* expression by affecting *GABA-A* receptors in early embryo development. In addition, at the time of ovulation in adults, a main double excitatory input to (GnRH) neurons is provided by the AVPV/PeN neuron population, leading to the expression of *GABA* and kisspeptin.

Another significantly associated SNP identified in the present study (rs268256209) is located 99,644 bp upstream of the *AKAP13* gene. *AKAP13* is a member of the *AKAP* family which is a structurally diverse protein and is involved in the binding process to the regulatory subunit of protein kinase A (PKA) and confining the holoenzyme to discrete locations within the cell. Luconi et al. (34) reported that *AKAP* proteins are expressed in both female and male reproductive systems, especially during gametogenesis. It has been suggested that *AKAP*–*PKA* interactions control the maturation of oocytes (35).

Based on the functional role of *PPP1R1C, SV2B, TRNAS-GCU* and *SSFA2* genes, there is no evidence to suspect a causative association with the LSB trait in our study. Whereas, both *GABRA5* and *AKAP13*, are the more likely genes potentially influencing LSB based on their influence of reproductive processes.

The results of least-square analyses showed that rs268288690 SNP leads to a significant increase in litter size in Markhoz goats (*p* < 0.01) so that goats having two copies of the mutated allele (GG genotype) had more kids within the litter than those carrying one or no copy of the mutated allele (AG and AA genotypes). The GG genotype of rs268258357 SNP indicated the highest litter size (1.822 ± 0.161) among all identified genotypes. Similarly, rs268267345 SNP also had a positive impact on litter size. These findings revealed that only rs268256209 SNP negatively affected litter size, while the other three SNPs identified from GWAS positively influenced the number of kids in Markhoz goats.

Estimated heritability for LSB and LSW was 0.018 and 0.32e−6, respectively. These values are lower than the estimated genomic heritability of 0.011 for LSB and 0.21e−7 for LSW. One of the main reasons for the observed differences could be different sample sizes used for estimating heritability *via* BLUP and LDAK models. In BLUP, we used all available data (3,410) and pedigree (5,396) records, while only 136 genotyped individuals were used in the LDAK method to predict genomic heritability. Besides, much of the heritability of traits may not be accounted for by rare, low-frequency genetic variants, known as the missing heritability problem (36).

There are many genes that have been identified as associated with litter size in goat and sheep. Among them, *GDF9, BMP15, BMPR1B, and IGF1* genes have been widely discussed in literature acknowledging their effects on litter size. However, to the best of our knowledge, the associations between candidate genes detected in the present study and litter size have not been reported previously. Therefore, according to their vital functions in reproductive processes, the *GABRA5* and *AKAP13* genes could be important novel candidate genes for litter size in small ruminants. However, our study had some limitations, including small sample size and relatively low heritability of studied traits. Thus, more genotyped animals are required to validate the impact of these potential candidate genes on litter size in goats.

Additionally, due to the high genomic correlation between LSB and LSW traits, we expected to find some common regions for the two traits, but the GWAS for the PBV for LSW failed to detect any significantly associated genomic regions. One possible reason could be that LSW is affected by environmental conditions or the pattern of effects may be altered by environment and genetic interactions. Furthermore, LSW may be influenced by rare causal variants that are not included in the current goat medium-density SNP-chip and not captured by linkage disequilibrium. Additionally, the complexity of gene interactions on this trait, such as epistasis, was not considered in this study and may play a more prominent role in regulation. The next possible reason could be the low frequency of the causal variants, which require a larger sample size to capture effect.

Despite the significant markers found for LSB, the present study has some limitations regarding LSW trait. It should be noted that LSB and LSW had extremely low heritabilities, suggesting a low possibility to achieve rapid genetic progress through phenotypic selection for LSB. Furthermore, LSW is generally connected to the mothering ability of the doe and environmental factors such as farm management. In addition, the sample size used in this study was limited, due to the low population size of Markhoz goats. Thus, caution must be taken when interpreting the results of the present GWAS, especially for LSW trait.

## Conclusion

To conclude, we found plausible candidate genes based on SNPs associated with the EBV of LSB in the Markhoz goats using the 50K Caprine SNP-chip for the first time. The significant SNPs and genes identified in the present study can be beneficial for future molecular-based breeding for increased litter size at birth in goats; however, due to the low sample size used in this study, the results should be interpreted with caution. It is noteworthy that a breeding program focused on the major variations for LSB would not necessarily increase the number of surviving progenies due to the extremely low heritability of LSW. There may be no net impact on LSW from the slight increase in litter size caused by the substantial variations due to reduced viability.

## Data availability statement

The SNP genotype data and EBVs for LSB and LSW are available in the Zenodo repository (https://zenodo.org/record/5824843).

## Ethics statement

The animal study was reviewed and approved by Cornell University Institutional Animal Care and Use Committee (protocol #2014-0121). Written informed consent was obtained from the owners for the participation of their animals in this study.

## Author contributions

PM performed data curation, formal analysis, visualization, methodology, and writing—original draft. AR and JR managed the project and contributed to writing— review and editing manuscript. AN-G conducted data curation, interpreted the results and contributed to review and editing manuscript. MR was responsible for data visualization. HH contributed to review and editing manuscript and provided SNP data and financial support. All authors contributed to the article and approved the submitted version.

## Funding

Funding for the Markhoz goats' genotyping and part of the analysis has been supported by the laboratory of HH at Cornell University.

## Conflict of interest

The authors declare that the research was conducted in the absence of any commercial or financial relationships that could be construed as a potential conflict of interest.

## Publisher's note

All claims expressed in this article are solely those of the authors and do not necessarily represent those of their affiliated organizations, or those of the publisher, the editors and the reviewers. Any product that may be evaluated in this article, or claim that may be made by its manufacturer, is not guaranteed or endorsed by the publisher.
